# Supplementary material for: It Works! Organic-Waste-Assisted Trichoderma spp. Solid-State Fermentation on Agricultural Digestate
Source: Microorganisms. 2022 Jan 13;10(1):164. doi: 10.3390/microorganisms10010164 (PMC8780502; doi:10.3390/microorganisms10010164)
Supplement: Supplementary file 1 [file microorganisms-10-00164-s001.zip › microorganisms-1532140-supplementary.pdf]

Table S1 Biomass of *Trichoderma* spp. content in each dose of the root elongation assay expressed as gram of mycelium.

	**Mycelium (g)**			
**Doses (%)**	***T. asperellum* R**	***T. atroviride* Ta13**	***T. harzianum* T-22**	***T. reesei* RUT-C30**
100	8.90	11.68	3.49	13.80
30	2.67	3.51	1.05	4.14
15	1.33	1.75	0.52	2.07
7.5	0.67	0.88	0.26	1.03
3.75	0.33	0.44	0.13	0.52
1.8	0.17	0.22	0.07	0.26
